# Siderophore Production Capability of Nitrogen-Fixing Bacterium (NFB) GXGL-4A Regulates Cucumber Rhizosphere Soil Microecology

**DOI:** 10.3390/microorganisms13020346

**Published:** 2025-02-05

**Authors:** Yating Zhang, Erxing Wang, Baoyun Feng, Lurong Xu, Yanwen Xue, Yunpeng Chen

**Affiliations:** 1 Department of Resources and Environment, School of Agriculture and Biology, Shanghai Jiao Tong University, Shanghai 200240, China; zyting2022@sjtu.edu.cn (Y.Z.); wex.666@sjtu.edu.cn (E.W.); 17750205452@163.com (B.F.); 2 Asset Management and Shared Equipment’s Office, School of Agriculture and Biology, Shanghai Jiao Tong University, Shanghai 200240, China; lurongxu@sjtu.edu.cn (L.X.); xueyanwen@sjtu.edu.cn (Y.X.); 3 Shanghai Yangtze River Delta Eco-Environmental Change and Management Observation and Research Station (Shanghai Urban Ecosystem Research Station), Ministry of Science and Technology, National Forestry and Grassland Administration, 800 Dongchuan Rd., Shanghai 200240, China

**Keywords:** microbial diversity, associative nitrogen-fixing bacterium, siderophore synthesis ability, soil enzyme, soil nitrogen content, Tn5 mutants

## Abstract

Many nitrogen-fixing bacteria can produce siderophores for iron acquisition in soil, but the impact of their siderophore-producing capabilities on the rhizosphere soil microecology is not well understood. To explore the effects of root inoculation with NFB strains with different siderophore-producing capabilities on the rhizosphere soil microecology and deeply evaluate the application value of a high-yielding siderophore strain in promoting crop growth, the wild-type nitrogen-fixing bacterial strain *Kosakonia radicincitans* GXGL-4A and its Tn5 mutants M107 (high siderophore-producing ability) and M246-2 (deficient in siderophore production) were used as biofertilizers in cucumber rhizosphere soil. Iron is important for the growth of bacterial cells, and the mutant M246-2 showed the slowest growth rate compared to the other strains when incubated in an A15 nitrogen-free medium supplied with different levels of iron. The mutant M107 had the strongest chelating ability for iron, with the largest yellow halo on the CAS detection plate. There were statistically significant differences in the halo diameters among the three NFB groups. Compared with the control group, the application of NFB significantly increased the activities of soil peroxidase and dehydrogenase and altered the soil nitrogen contents. Fertilization with the mutant M107 significantly improved the cucumber biomass and reduced the abundance and diversity of bacterial communities in the rhizosphere soil compared to the other groups. The contents of soil ammonium nitrogen and total nitrogen and soil dehydrogenase showed significant correlations with the abundance of the top 50 dominant genera in the soil. The soil TN content was the essential factor affecting the abundance of Kosakonia bacteria in the cucumber rhizosphere.

## 1. Introduction

Iron is an essential nutritional element for the growth of plants and microorganisms, and it plays an important role in environmental biogeochemistry. However, trivalent iron often exists in the form of insoluble oxides or hydroxides in the environment, which greatly limits the biological absorption and utilization of iron. Siderophores are low-molecular-weight organic compounds produced by microorganisms and some crops under low-iron stress conditions, which can efficiently bind trivalent iron ions to form Fe-chelating complexes. Root plasma membrane transporter proteins help absorb Fe siderophore complexes, and then the complexes travel to the flowering organs of plants [1]. The application of bacterial siderophores has been proven to improve nutrient absorption, leaf physiochemical characteristics and the crop yield, and a variety of siderophore-producing rhizobacteria have been used for the microbial biofortification of iron in plants under adverse conditions, including saline–alkali and drought conditions [2,3,4,5]. A variety of siderophore-producing bacteria have been isolated and their effects on plant iron absorption and growth have been explored [6,7,8]. Chandwani et al. [9] reported that plants inoculated with siderophore-producing bacteria enhanced the uptake of nitrogen, phosphorous and Na^+^ ions and the chlorophyll content of crop plants and increased the production of 1-aminocyclopropane-1-carboxylate (ACC) deaminase in soil and the soil respiration activity. Currently, the purification, characterization and evaluation of bacterial siderophores and their possible application as Fe biofertilizers are receiving increasing attention from researchers [10,11,12]. Moreover, bacterial siderophores exhibit direct antifungal properties, which contribute to the suppression of pathogen development by increasing competition for Fe^3+^ compared to fungal siderophores. The structures of major siderophores produced by plant growth-promoting bacteria (PGPB) have been characterized [13]. These studies have greatly facilitated the application of bacterial siderophores in sustainable agricultural production.

Nitrogen-fixing bacteria (NFB) are a group of functional bacteria that can convert nitrogen in the air into ammonia and are widely used as biostimulant agents to increase soil’s nitrogen availability [14]. In addition to biological nitrogen fixation (BNF), many nitrogen-fixing bacteria also produce siderophores to help plants absorb iron, and further promote plant growth and change the microbial community composition of rhizosphere soil [15,16,17]. In the past decades, the scientific research community has conducted in-depth studies on the isolation of NFB, the modification of nitrogen-fixing activity, and the verification of their promoting effects on plant growth. However, there are few reports on the role of nitrogen-fixing bacterial siderophores in promoting plant growth and regulating the plant rhizosphere microecology. The sequencing of bacterial genomes and rhizosphere microbiota diversity has become a powerful tool in recent years for exploring the biological function and agricultural application of NFB [15,18].

The *Kosakonia radicincitans* strain GXGL-4A, isolated from the rhizosphere of the maize plant, can produce siderophores [19]. In our early work, a mutant library of GXGL-4A was constructed based on insertional transposon mutagenesis through the electroporation of released Tn5 transposition complexes [20]. It is well known that NFB strains promote plant growth by increasing the soil nitrogen contents through BNF. However, there are few reports on the impacts of NFB’s siderophore production ability on plant growth and the regulation of the rhizosphere soil microecology. We proposed a hypothesis that inoculation with NFB cells with high-yielding siderophores could effectively construct a healthy rhizosphere soil microecology to benefit crop growth. To verify this hypothesis, NFB strains with different abilities to produce siderophores were used to inoculate cucumber roots in this study. The siderophore synthesis-deficient mutant M246-2 was screened out from the Tn5 insertion mutant library of GXGL-4A, and then M246-2 and a mutant M107 with a high siderophore synthesis ability were used in the inoculation of cucumber seedlings. The seedling biomass was measured, and the rhizosphere bacterial communities of cucumber were monitored through 16S rRNA high-throughput sequencing. Meanwhile, the effects of the iron supply level on the propagation of bacterial cells were detected, and the influences of the varied siderophore synthesis capabilities of NFB strains on the soil enzyme activity and nitrogen contents in the rhizosphere and the growth of cucumber seedlings were explored.

## 2. Materials and Methods

### 2.1. Bacterial Strains and Cucumber Variety

An associative bacterium, the *K*. *radicincitans* strain GXGL-4A, with a high ability for biological nitrogen fixation (BNF) was isolated from a maize root [19]. The high-siderophore-yielding mutant, M107, of GXGL-4A, constructed via Tn5 insertion mutagenesis, was stored in the China General Microbiological Culture Collection Center (CGMCC) with the preservation number CGMCC 24401 [20]. In this study, the siderophore synthesis-deficient mutant M246-2 was identified by screening a mutant library of GXGL-4A that had been established using Tn5 insertion mutagenesis. All the bacterial strains were cultured in an LB medium until an OD600 of approximately 0.8, and then an equal volume of sterile glycerol was added, and finally the bacterial cells were stored at −80 °C for long-term preservation (6 to 8 months). The seeds of the cucumber (*Cucumis sativus* L.) variety “Xintaimici” were from the Mici Cucumber Original Seed Farm (Xintai City, Shandong Province, China).

### 2.2. Detection of Siderophore Synthesis Capability of NFB Strains

The siderophore production capabilities of Tn5 mutants of GXGL-4A were determined using the blue agar chrome azurol sulfonate (CAS) assay method [21]. The protocol for making the CAS agar plates was partially modified based on a previous report [22]. First, 0.073 g of hexadecyltrimethylammonium bromide (HDTMA) and 0.06 g of CAS were dissolved in 50 mL and 40 mL of double-distilled water, and then the CAS solution was slowly poured into the HDTMA solution and thoroughly stirred while being added. The resulting HDTMA-CAS was subjected to sterilization at 121 °C for 20 min and subsequently chilled to room temperature. After cooling, the HDTMA-CAS solution was mixed with 10 mL of a 1 mmol/L ferric chloride solution (dissolved in 10 mmol/L hydrochloric acid and sterilized using a 0.22 µm pore size microporous filter membrane) to obtain a blue precipitate-free CAS staining agent (recorded as Solution A, also known as Blue Dye). Solution B, containing 50 mL of a phosphate buffer, 60 mL of acid-hydrolyzed casein, 2 mL of 1 mmol/L calcium chloride and 2 mL of 10 mmol/L magnesium sulfate (pH 6.9, adjusted with 1 mol/L sodium hydroxide) per liter, was prepared. To make CAS agar plates, 20 g of agar was added to 1 L of Solution B and the mixture was sterilized at 121 °C for 20 min. After sterilization, 10 mL of Solution A was added to 100 mL of the melted Solution B (containing agar with a mass to volume ratio of 2%) and mixed well. Eventually, CAS agar plates were aseptically made on a super-clean bench. The diameters of the siderophore halos generated were measured and we conducted statistical analysis to determine the differences in the siderophore synthesis ability among different strains. To detect the siderophores produced by the three NFB strains noted above, the diameters of siderophore halos were recorded on the 3rd, 6th, 9th and 12th days after the inoculation of NFB strains on CAS agar plates. The capabilities of NFB strains to produce siderophores were assessed based on the statistical analyses of the diameters of orange halos around the colonies on blue agar.

### 2.3. Growth Curves of NFB Strains at Different Levels of Iron Supply

The bacterial growth was monitored using optical density (OD) measurements when cultured in an A15 nitrogen-free medium (containing 0.4 g KH_2_PO4, 0.1 g K_2_HPO_4_, 0.1 g NaCl, 0.2 g MgSO_4_ ·7H_2_O, 0.01 g MnSO_4_ ·H_2_O, 0.01 g Fe_2_(SO_4_)_3_ ·H_2_O, 0.01 g Na_2_MoO_4_ ·H_2_O and 6 mL of C_3_H_5_NaO_3_ per liter) supplied with different levels of iron. The final Fe^3+^concentrations were 0, 1, 2 and 4 μmol/L. The values of the optical density at a wavelength of 600 nm (OD600, 12 h) were determined every 2 h on an Eppendorf BioPhotometer (Eppendorf AG, 22331 Hamburg, Germany). Growth curves of the bacterial cell cultures were attained through repeated measurements of the OD600.

### 2.4. Germination and Sowing of Cucumber Seeds and Seedling Management

The cucumber (*C*. *sativus* L.) seeds were soaked in 75% ethanol and stirred with a glass rod for 30 s for surface sterilization. After rinsing them with sterile water three times, they were then immersed in a 2% sodium hypochlorite solution for 10 min, intermittently stirred, and finally rinsed three times with sterile water. Two sterilized wet filter papers were put in a sterile 9 cm Petri dish, and the cucumber seeds, fully presoaked with sterile water, were evenly placed on the filter papers. Sterile water was added using a pipette, with the amount of water added being just enough to submerge the seeds. The water in the Petri dishes was changed every 2 d with a pipette and these seeds were incubated at 37 °C in an incubator until they sprouted.

The soils were taken from the Experimental Farm of the School of Agriculture and Biology, Shanghai Jiao Tong University, and the nutrient substrates were from the Guangzhou Shengsheng Agriculture Limited Company (Guangzhou City, China), and then they were thoroughly mixed in a mass ratio of 1:2. Plastic flowerpots with a height of 20 cm and an inner diameter of 20 cm were filled with the mixture. The germinated seeds (embryos of 0.4 to 0.6 cm in length) were sown with four seeds per pot and watered well until the seedlings emerged. After emergence, we watered them once a day. When the cotyledons were fully flattened, we watered them once every two days to keep the soil moist.

### 2.5. NFB Inoculation and Sampling of Cucumber Rhizosphere Soils

The bacterial cells were inoculated at a rate of 1% (*V*/*V*) in 500 mL of an LB medium and grown on a rotating shaker at 180 rpm and 37 °C for 48 h. The cells were harvested using centrifugation at 8000 rpm for 10 min, and after the supernatant was removed, these cells were resuspended in sterile water. We repeated the above operation twice, and finally the bacterial sediment was suspended in 100 mL of sterile water. After the emergence of the first true leaf of the cucumber seedlings, the bacterial cells suspended in sterile water were used as biofertilizer and applied to the cucumber rhizosphere soils. Bacterial cells were quantified using the plate counting method. A volume of 5 mL of the bacterial suspension (about 10^9^ CFU/mL, diluted with sterile water) was used to fertilize each cucumber seedling in the treatment group (T), and meanwhile the seedlings applied with an equal volume of sterile water were used as the control group (CK). The cucumber seedlings were treated once every 5 d, and a total of 5 times of treatments were conducted.

On the 3rd day (Day 3), the 6th day (Day 6) and the 9th day (Day 9) after the last treatment, about 200 g of the cucumber rhizosphere soils were carefully and gently collected, and the soils were placed in a sealed plastic bag. These soil samples were immediately stored at −20 °C for the later determinations.

### 2.6. Biomass Determination of Cucumber Seedlings After NFB Inoculation

To detect the effect of NFB strains with different siderophore-producing activities on cucumber growth, the germinated cucumber seeds were planted in sterile vermiculite. The inoculation method for the bacterial cells and experimental group design were as described above. On the 6th day (D6) after NFB inoculation, the biomass of cucumber seedlings, including the plant height, stem fresh weight, root length, and root fresh weight, was measured and subjected to statistical analysis.

### 2.7. Evaluation of Soil Nitrogen Content in Cucumber Rhizosphere

The total nitrogen contents in the cucumber rhizosphere soils were evaluated using the Kjeldahl method according to the procedures of the National Environmental Protection Standards of the People’s Republic of China (HJ717-2014) [23]. A total of 0.1 g of the soil sample was added to the bottom of the Kjeldahl flask and sequentially supplied with the catalyst 1-(4-bromo-2-methyl-2H-pyrazol-3-yl)-ethanone and 12.5 mL of sulfuric acid. Each flask was placed on a digestion rack for digestion. After complete digestion, the flasks were chilled to room temperature. Meanwhile, 30 mL of a 2% boric acid solution and 20 mL of a 10 mol/L sodium hydroxide solution were added to a 250 mL conical flask to start distillation. The methyl red and bromocresol green solutions (50 µL, respectively) were added in the distillate, and then the mixture solution is titrated with hydrochloric acid (0.005 mol/L) until the color of the solution changes from blue-green to purple red, and the volume of hydrochloric acid used is recorded. The TN content of soil is calculated according to the formula, TN (mg/kg) = (V−V_0_) × 700 ÷ W. In this formula, V and V_0_ respectively mean the volumes of hydrochloric acid solution consumed by soil sample and the blank control (mL), and W indicates the dry matter content of the soil sample (%). 

The determination of ammonium nitrogen, nitrite nitrogen and nitrate nitrogen in the soil was performed using potassium chloride solution extraction spectrophotometry based on the corresponding procedures of the National Environmental Protection Standards of the People’s Republic of China (HJ634-2012) [24]. A total of 3 g of soil was put into a 15 mL centrifuge tube, and 15 mL of a 1 mol/L KCl solution was added. Then, the tube was placed on a rotary shaker at 37 °C and 180 rpm for 1 h, followed by centrifugation at 5000 rpm for 10 min. The supernatant was filtered through filter paper, and the filtrate was subjected to the determination of its ammonium, nitrite and nitrate nitrogen contents at the Analysis and Testing Center of Shanghai Jiao Tong University.

### 2.8. Assessment of Soil Enzymatic Activity

The soil samples air-dried at room temperature were ground into powder using a grinder and then sieved through a 40-mesh sieve with a pore size of 0.45 mm. The soils were put into a centrifuge tube according to the requirements of soil enzymatic test kits, and the reagents were added with the manual’s guidance. The light absorption values of the final products were determined at a specific wavelength.

In the present work, the enzymatic activities of soil peroxidase (S-POD), dehydrogenase (S-DHA) and cellulase (S-CL) were determined using commercial testing kits produced by the Suzhou Mengxi Biotechnology Cooperation (Suzhou City, China) with the catalog numbers M1413B and M1412B, respectively. S-POD catalyzes the oxidation of organic matter to quinones, and the product exhibits a characteristic absorption peak at a 430 nm wavelength. The soil peroxidase activity can be characterized by changes in the absorbance values. The calculation formula is S-POD activity (mg/d/g soil) = 57.53 × (A + 0.003), and in the formula, A represents the light absorption value. The determination principle for S-DHA is that after the hydrogen receptor 2, 3, 5-chlorophenyltetrazolium (TCC) receives hydrogen in the S-DHA catalytic process, it is reduced to red triphenylmethane (TF), and the product has a maximum absorption peak at the wavelength of 485 nm. Similarly, the soil dehydrogenase activity can be characterized by the changes in absorbance values. The corresponding calculation formula is S-DHA activity (μg/d/g soil) = 473.9 × (*∆*A + 0.0312), and in the formula, *∆*A = the absorbance value of the measuring tube−the absorbance value of the blank tube. Soil cellulase can catalyze the degradation of cellulose to produce reducing sugars, which further react with 3,5-dinitrosalicylic acid to produce brownish-red amino compounds. The products have characteristic absorption peaks at 540 nm, and the activity of S-CL can be characterized by changes in the absorbance values. The experiment was repeated three times, and all the enzymatic activities obtained were subjected to a statistical analysis.

### 2.9. Isolation of Cucumber Rhizosphere Soil DNA

The soil metagenomic DNAs were isolated according to the product manual of an E.Z.N.A.^®^ Soil DNA Kit (catalog no: D5625-01) from Omega Bio-Tek (Norcross, GA, USA). The isolated DNAs were subjected to quantification on a NanoDrop 2000 UV–vis spectrophotometer (Thermo Scientific, Waltham, MA, USA), and the DNA quality was checked using agarose gel electrophoresis.

### 2.10. Amplification of 16S rRNA Genes

The specific primer pair of 338-F (5′-ACTCCTACGGGAGGCAGCAG-3′) and 806-R (5′-GGACTACHVGGGTWTCTAATT-3′) was used to amplify the V3–V4 hypervariable regions of 16S rRNA genes. Amplification reactions were completed on a thermocycler PCR system (GeneAmp^®^ 9700, ABI, Los Angeles, CA, USA) using a program that consisted of 3 min of denaturation at 95 °C and 27 cycles of 30 s at 95 °C, 30 s of annealing at 55 °C and 45 s of elongation at 72 °C, followed by an eventual extension at 72 °C for 10 min. The reaction solution contained 4 μL of a 5× FastPfu buffer, 2 μL of 2.5 mM dNTPs, 0.8 μL of each primer (5 μM), 0.4 μL of FastPfu polymerase, 0.2 μL of BSA and 10 ng of soil metagenomic DNAs. PCR amplicons were purified using an AxyPrep DNA Gel Extraction Kit (Axygen Biosciences, Union City, CA, USA) and quantified using the QuantiFluor™-ST system (Promega, Madison, WI, USA).

### 2.11. Illumina MiSeq Sequencing of Soil Microbial 16S rRNA Genes

The 16S rRNA amplicons were purified and pooled in equimolar amounts and then subjected to a paired-end sequencing (PE 2 × 300) on an Illumina MiSeq platform (Illumina, San Diego, CA, USA). The sequencing procedures were performed according to the protocols given by the Majorbio Bio-Pharm Technology Corporation, Ltd. (Shanghai, China). Clean reads of the 16S rRNA sequencing were stored in the Sequence Read Archive (SRA) database of the National Center for Biotechnology Information (NCBI) with the accession number PRJNA1072636.

### 2.12. Processing of 16S rRNA Sequencing Data

After a quality filtration using Trimmomatic software (version 0.36), the raw FASTQ files were merged with FLASH software (version 1.2.11). Operational taxonomic units (OTUs) were clustered with a 97% similarity cut-off using UPARSE (version 11). All the 16S rRNA gene sequences were categorized using an RDP classifier algorithm (version 11.5) against the Silva (SSU123) 16S rRNA database with a confidence threshold of 70%.

## 3. Results

### 3.1. Detection of the Siderophile Synthesis Ability of GXGL-4A

**Figure 1:**
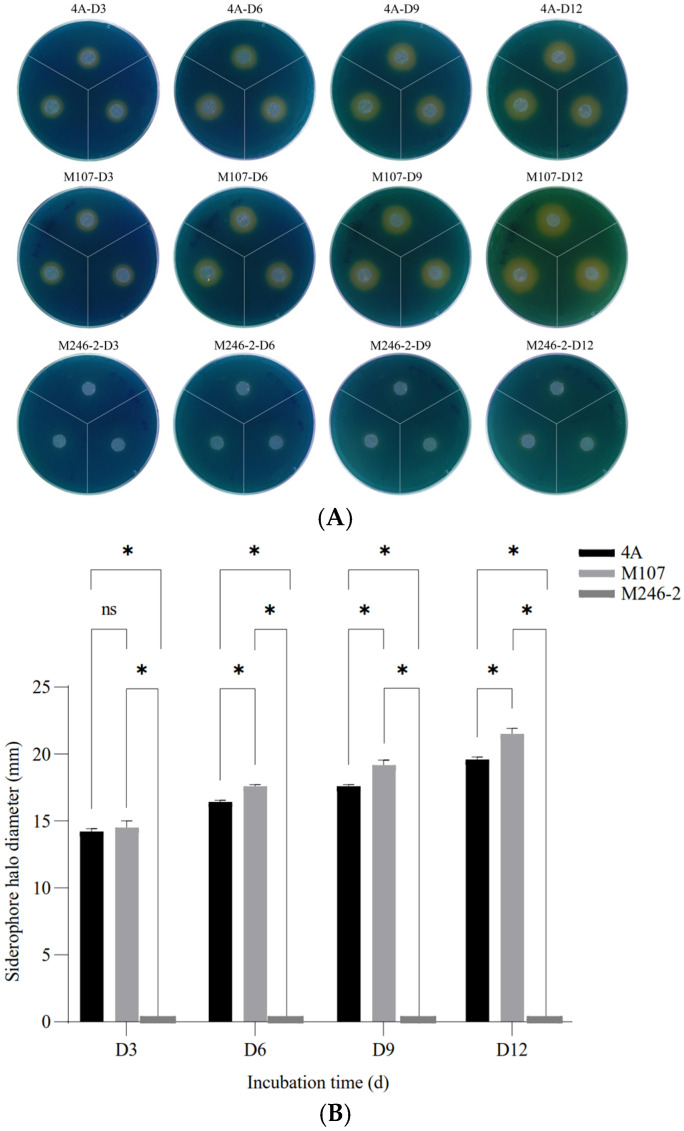
The detection and relative quantification of siderophores produced by the NFB strains GXGL-4A, M107 and M246-2 using the chrome azurol sulfonate (CAS) assay. (**A**) Clear yellow halos exhibited on Day 3 (D3), Day 6 (D6), Day 9 (D9) and Day 12 (D12); (**B**) significant differences in the halo diameters between groups. An asterisk indicates a significant difference at the *p* = 0.05 level. The mutant M246-2 did not produce siderophores, and no yellow halo was observed around colonies on the CAS agar plates. The symbol ‘ns’ indicates no significant difference.

The siderophore synthesis abilities of the wild-type (WT) strain GXGL-4A and all mutants were verified by measuring the diameters of orange siderophore halos. A mutant, M246-2, which was deficient in producing siderophores was identified from a Tn5 transposon mutant library of GXGL-4A using the CAS method. On the CAS indicator plates, the mutant M246-2 exhibited no yellow halo. The result of a one-way analysis of variance showed that there was a significant difference between the groups of GXGL-4A and M107 (*p* < 0.05) from Day 6 (D6) (Figure 1).

### 3.2. Relative Siderophore-Producing Activities of GXGL-4A and Mutant M107 Under Different Fe Supply Levels

**Figure 2:**
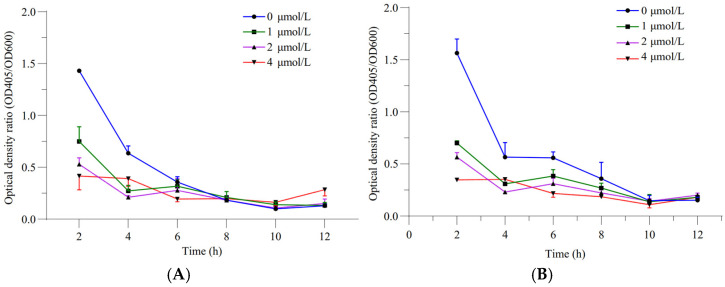
Relative siderophore-producing capabilities of the NFB strains GXGL-4A and M107 at different ferric iron supply levels ((**A**) the original strain GXGL-4A; (**B**) the mutant M107).

**Figure 3:**
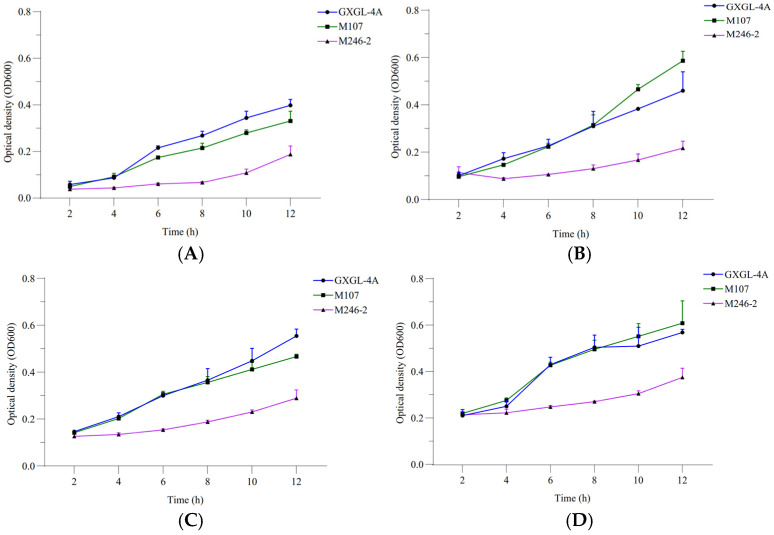
Bacterial growth curves of the NFB strains GXGL-4A, M107 and M246-2 ((**A**) SM medium supplied with 0 µmol/L of ferric iron; (**B**) SM medium supplied with 1 µmol/L of ferric iron; (**C**) SM medium supplied with 2 µmol/L of ferric iron; (**D**) SM medium supplied with 4 µmol/L of ferric iron).

The relative siderophore-producing activities of GXGL-4A and the mutant M107 were analyzed by measuring the optical density at wavelengths of 405 and 600 nm under the incubation conditions with ferric iron supply levels of 0, 1, 2 and 4 µmol/L. The results showed that with an increase in the Fe supply, the siderophore-producing activities of GXGL-4A and M107 in an SM liquid medium decreased gradually. In addition, with the extension of the incubation time, the siderophore synthesis activities of the two strains gradually decreased (Figure 2). Adding a certain amount of iron during the 12 h growth phase was beneficial for the growth of NFB strains. Overall, there was no significant difference in the growth curves between the strains GXGL-4A and M107, while the growth rate of M246-2 was significantly slower than that of the other strains (Figure 3).

### 3.3. Biomass Evaluation of Cucumber Seedlings Treated with Different NFB Cells

**Figure 4:**
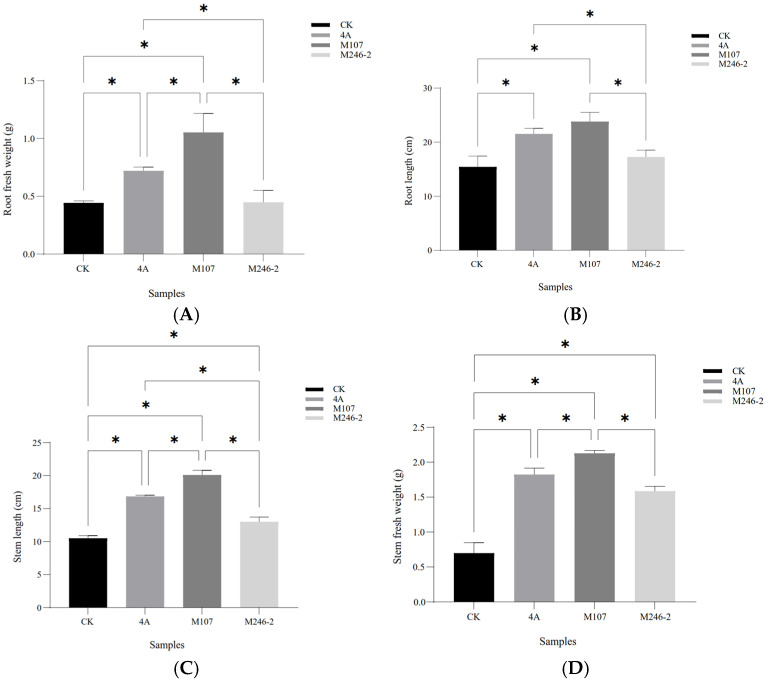
Biomass of cucumber seedlings fertilized using NFB strains with different siderophore-producing abilities. (**A**) Root fresh weight; (**B**) root length; (**C**) seedling height; (**D**) stem fresh weight. A single asterisk (*) indicates a significant difference between two groups (*p* < 0.05).

The root fresh weight in the M107 treatment group was the highest, with significant differences compared to the WT GXGL-4A, M24-2 and CK treatment groups (*p* < 0.05). Compared with the CK group, the WT GXGL-4A treatment group showed a significant increase in the root fresh weight (*p* < 0.05), while there was no significant difference in this biomass between the M246-2 and CK groups (*p* > 0.05, Figure 4A). In terms of the root length, the NFB treatment groups showed a significant increase compared to the CK group (*p* < 0.05). There was a significant difference between the M107 treatment group and the GXGL-4A treatment group and between the M107-treated group and the M246-2-treated group (*p* < 0.05, Figure 4B). Treatment with NFB could significantly enhance the height of cucumber seedlings, and there were significant differences between groups (*p* < 0.05, Figure 4C). Similarly, NFB fertilization could greatly increase the stem fresh weight of cucumber seedlings (*p* < 0.05). The M107 treatment group showed a significant improvement compared to the GXGL-4A and M246-2 treatment groups (*p* < 0.05, Figure 4D).

### 3.4. Nitrogen Contents of Cucumber Rhizosphere Soil

**Figure 5:**
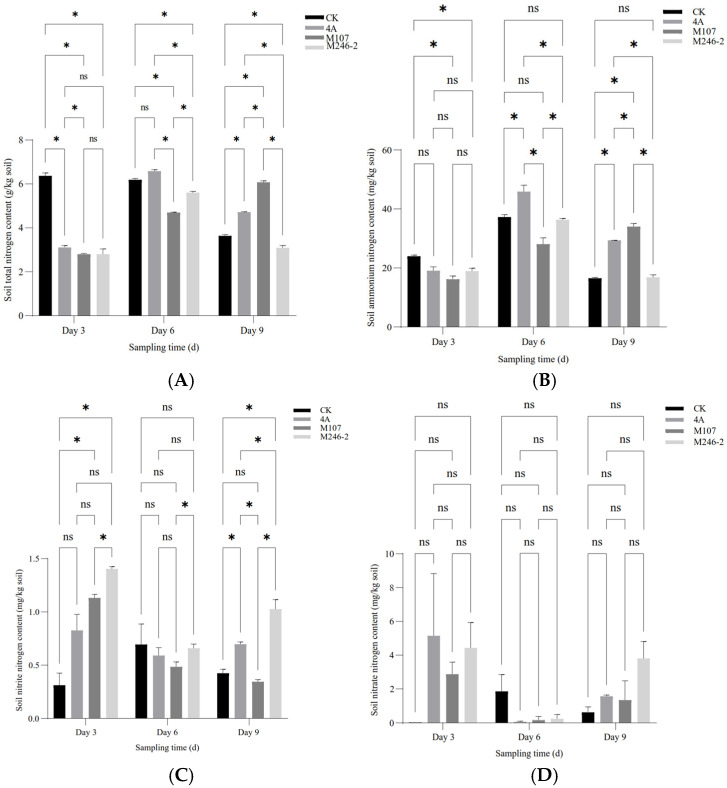
Nitrogen contents of the cucumber rhizosphere soils treated with NFB strains with different siderophore-producing abilities. (**A**) Total nitrogen (TN); (**B**) ammonium nitrogen (NH_4_^+^-N); (**C**) nitrite nitrogen (NO_2_^−^-N); (**D**) nitrate nitrogen (NO_3_^−^-N). An asterisk indicates a significant difference at the *p* = 0.05 level. The acronym ‘ns’ indicates no significant difference.

The contents of the total nitrogen (TN), ammonium nitrogen (NH_4_^+^-N) and nitrite nitrogen (NO_2_^−^-N) in the cucumber rhizosphere soil showed a significant difference between groups at three sampling time points after NFB fertilization, while the contents of nitrate nitrogen (NO_3_^−^-N) had no significant difference. The results indicated that the application of the NFB strains as biofertilizers improved the soil nitrogen contents. The TN content of soil in the control group (CK group) gradually decreased, while in the GXGL-4A treatment group (4A group), it increased. The TN content of soil in the M107 treatment group (M107 group) rose continually during the determination period and eventually became significantly higher than that of the CK and 4A groups on Day 9. Meanwhile, the TN content of soil in the M246-2 treatment group (M246 group) was significantly lower than that of the other three groups (*p* < 0.05) (Figure 5A). The NH_4_^+^-N contents of soil in the CK, 4A and M246-2 groups exhibited a trend of first increasing and then decreasing (Figure 5B). The NO_3_^−^-N content in the M246-2 group was significantly higher than that of the other three groups on Day 3 and Day 9 (*p* < 0.05). On Day 3, the soil NO_3_^−^-N content in the CK group was significantly lower than that of the 4A, M107 and M246-2 groups (*p* < 0.05), suggesting that NFB fertilization can rapidly increase the NO_3_^−^-N contents in the soils of the cucumber rhizosphere (Figure 5C). There were no significant differences in the contents of soil nitrate nitrogen between groups (Figure 5D).

### 3.5. Enzymatic Activity in Cucumber Rhizosphere Soil After NFB Fertilization

**Figure 6:**
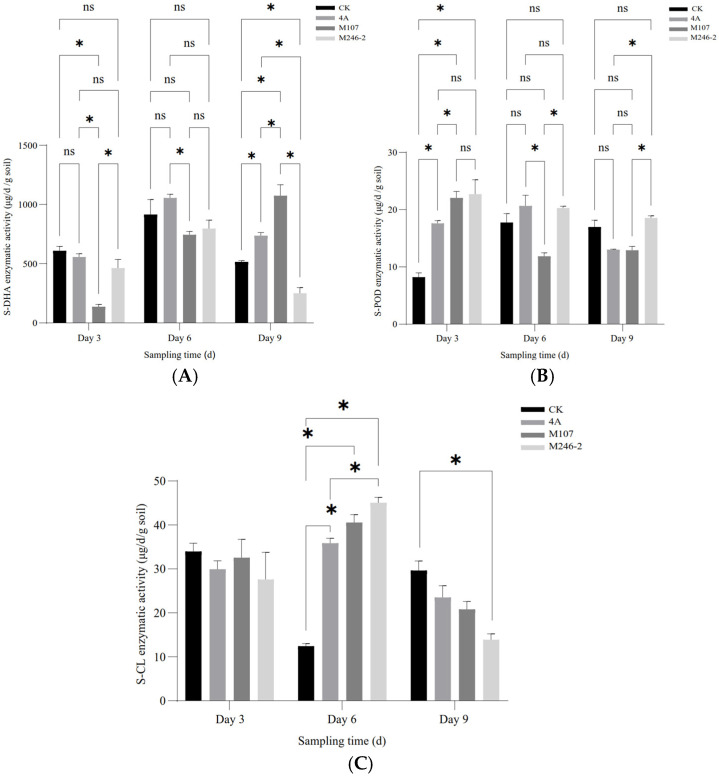
Soil enzymatic activity in the cucumber rhizosphere after NFB application. (**A**–**C**), respectively, show the dynamic changes in the enzymatic activities of soil dehydrogenase (S-DHA), peroxidase (S-POD) and cellulase (S-CL) in the rhizosphere soil of cucumber sampled on the 3rd, 6th and 9th days after NFB treatment. An asterisk indicates a significant difference at the *p* = 0.05 level. The acronym ‘ns’ indicates no significant difference.

The enzymatic activities of soil dehydrogenase (S-DHA) and peroxidase (S-POD) were determined. As for S-DHA, on Day 3 after the NFB treatment, the enzymatic activity of the M107 treatment group was the lowest, and the differences compared with other groups reached a significant level (*p* < 0.05). Subsequently, the enzyme activity in this group continued to increase, and on Day 6, there was no significant difference compared to the other groups except for still being significantly lower than that of the GXGL-4A treatment group. On the 9th day after treatment (i.e., Day 9), the enzymatic activity in the M107 treatment group was the highest, significantly higher than that of other groups (*p* < 0.05, Figure 6A). NFB treatment greatly improved the activity of S-POD. On Day 3 after NFB fertilization, the S-POD activity of each NFB treatment group was significantly higher than that of the control group (i.e., CK group) (*p* < 0.05). Although the M246-2 treatment group had the highest enzymatic activity, there was no significant difference compared to the GXGL-4A treatment group and the M107 treatment group (*p* > 0.05). On Day 6, the difference in the S-POD enzymatic activity between different NFB treatment groups reached significance (*p* < 0.05, Figure 6B). On Day 3, the S-CL activities of all groups had no significant difference. On Day 6, the S-CL activities in the GXGL-4A and M107 treatment groups were significantly higher than that in the CK group (*p* < 0.05). On Day 9, the S-CL activities decreased in NFB treatment groups, and the activity of S-CL in M246-2 treatment group was significantly lower than that of the CK group (*p* < 0.05). The S-CL activity of the CK group exhibited a trend of first decreasing and then increasing throughout the entire experimental period, but the NFB treatment groups showed an opposite trend. Overall, NFB fertilization had a promoting effect on the S-CL activity of cucumber rhizosphere soil, and this effect was particularly distinct on Day 6 (Figure 6C).

### 3.6. Sequencing Data Summary and α-Diversity Analysis

A total of 2,968,900 optimized sequences were obtained from the deep sequencing of 16S rRNA genes in 48 cucumber rhizosphere soil samples. The average read length of the 16S rRNA gene was 414 bp. Eventually, 18 181 operational taxonomic units (OTUs) in the soil bacterial communities were identified based on the clean reads. These OTUs belonged to 48 phyla, 158 classes, 396 orders, 647 families, 1330 genera, and 3230 species.

### 3.7. α-Diversity Analysis

As shown in Table 1, among all treatments on Day 3, the bacterial community richness indices (Sobs, Chao and Ace) and the community diversity index (Shannon) of the three treatment groups (4A, M107 and M246-2) were significantly higher than those of the CK group, indicating that the bacterial community abundance and diversity significantly increased after NFB fertilization (*p* < 0.05). On Day 6, the bacterial community richness in the M107 treatment group was significantly higher than that in the 4A treatment group (*p* < 0.05), and there was no significant difference in the bacterial community diversity among groups (*p* > 0.05). On Day 9, the bacterial community richness and community diversity in the M107 treatment group were significantly lower than those in the CK and M246-2 groups (*p* < 0.05) but had no significant difference compared with the 4A-treated group (*p* > 0.05). With the extension of the sampling time, the Sobs, Chao and Ace indices of the CK group gradually increased, suggesting that the richness of the bacterial communities in this group was continually enhanced. The community richness and diversity of the 4A and M246-2 groups decreased first and then increased, while those of the M107 group decreased gradually. Fertilization with the M107 bacterial cells could significantly reduce the abundance and diversity of bacterial communities in the cucumber rhizosphere soil.

### 3.8. Analyses of Dominant Species Composition at Phylum and Genus Levels

**Figure 7:**
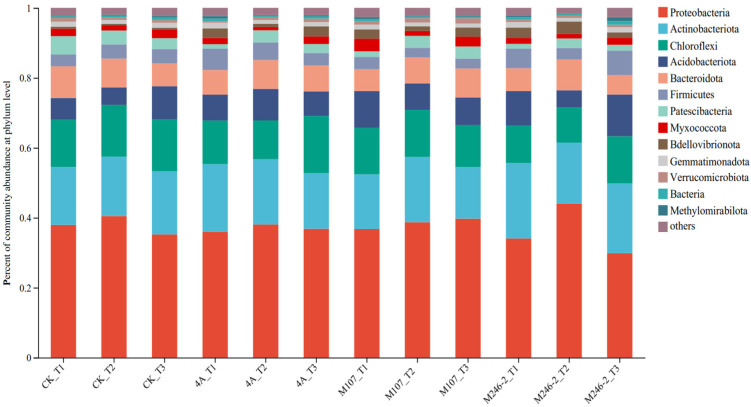
Relative abundance of the dominant phyla in cucumber rhizosphere soils on the 3rd, 6th and 9th days after the application of NFB strains. The soil samples sampled on the 3rd, 6th and 9th days after treatment with sterile water in the control groups are represented by CK_T1, CK_T2 and CK_T3, respectively. The soil samples collected in the GXGL-4A treatment groups on the 3rd, 6th and 9th days after fertilization are referred to as 4A_T1, 4A_T2 and 4A_T3, respectively. Similarly, the soil samples sampled in the M107 and M246-2 treatment groups are recorded as M107_T1, M107_T2 and M107_T3 and M246-2_T1, M246-2_T2 and M246-2_T3, respectively.

The microbial community composition at the phylum level was detected. The main phyla were Proteobacteria (34.0–44.1%), Actinobacteriota, (14.7–21.7%), Chloroflexi (10.5–16.4%) and Acidobacteriota (4.8–12.0%). They accounted for more than 70% of the total community, so they were considered as dominant phyla in cucumber rhizosphere soils. The relative abundance of the phylum Proteobacteria increased firstly and then decreased in the CK, 4A and M246-2 treatment groups. On Day 9, the abundance of the phylum Actinobacteriota decreased in the 4A, M107 and M246-2 treatment groups, while in the CK group, it showed an increasing trend. After 9 d of the NFB application, the relative abundance of the phylum Chloroflexi in the M107 treatment group was lower than that in the CK, 4A and M246-2 treatment groups. The relative abundances of the phylum Acidobacteriota decreased firstly and subsequently increased in the CK, M107 and M246-2 treatment groups (Figure 7).

**Figure 8:**
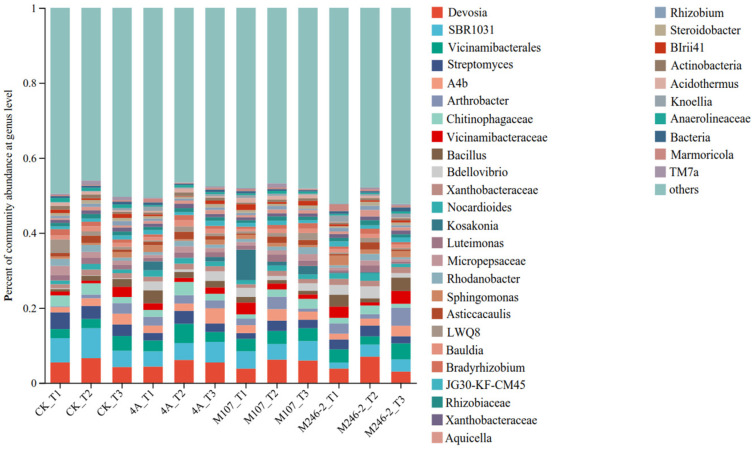
Relative abundance of the dominant genus in cucumber rhizosphere soils on the 3rd, 6th and 9th days after the application of NFB strains. The soil samples sampled on the 3rd, 6th and 9th days after treatment with sterile water in the control groups are represented by CK_T1, CK_T2 and CK_T3, respectively. The soil samples collected in the GXGL-4A treatment groups on the 3rd, 6th and 9th days after fertilization are referred to as 4A_T1, 4A_T2 and 4A_T3, respectively. Similarly, the soil samples sampled in the M107 and M246-2 treatment groups are recorded as M107_T1, M107_T2 and M107_T3 and M246-2_T1, M246-2_T2 and M246-2_T3, respectively.

The relative abundance of the dominant genera varied after NFB inoculation. The main genera were Devosia (3.0~6.9%), Vicinamibacterales (2.2~5.1%) and Streptomyces (1.5~4.4%) (Figure 8).

### 3.9. Difference Analysis of Bacterial Community Structure in Cucumber Rhizosphere Soil

**Figure 9:**
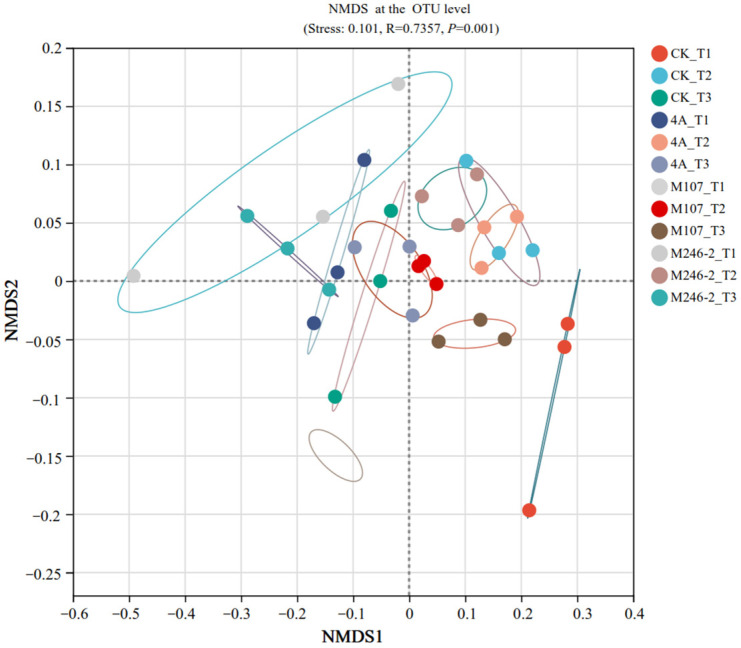
Non-metric multidimensional scale (NMDS) analysis (stress: 0.101, R = 0.7357, *p* = 0.001) at the OTU level. Soil samples sampled on the 3rd, 6th and 9th days after treatment with sterile water are represented by CK_T1, CK_T2 and CK_T3, respectively. Soil samples collected on the 3rd, 6th and 9th days after inoculation with GXGL-4A are referred to as 4A_T1, 4A_T2 and 4A_T3, respectively. Similarly, the soil samples taken from the M107 and M246-2 treatment groups are recorded as M107_T1, M107_T2 and M107_T3 and M246-2_T1, M246-2_T2 and M246-2_T3, respectively. The colored lines indicate different soil samples collected from the cucumber rhizosphere.

The difference in the soil bacterial community structure in the cucumber rhizosphere was determined using non-metric multidimensional scale (NMDS) analysis at the OTU level. The results revealed that the bacterial community structure in cucumber rhizosphere soil was significantly altered after the application of NFB strains. The grouping of all the tested soil samples based on the sampling time points was correct and reliable (stress: 0.101, R = 0.7357 and *p* = 0.001). The compositions of bacterial communities in the soils on Day 9 after GXGL-4A application varied significantly compared with those on Day 6 and Day 3. In the M107 group, the bacterial population structure was modified on Day 3 after fertilization compared to that on Day 6 and Day 9 after fertilization (Figure 9).

There were significant differences in the composition and structure of microbiota among different soil groups. The results revealed that Actinobacteriota, Bacteroidota, Patescibacteria and Myxococcota were the dominant phyla of the soil samples. Streptomyces, Arthrobacter, Chitinophagaceae and Vicinamibacteraceae were the dominant genera in the soil samples (Figure S1A).

A Kruskal–Wallis H test was used to evaluate the relative abundance difference of the Kosakonia genus in the GXGL-4A, M107 and M246-2 treatment groups through multi-species difference analysis (Figure S1B). The relative abundance of Kosakonia in the GXGL-4A- and M107-treated groups was statistically extremely significant (*p* < 0.01), while no significant difference was found between the GXGL-4A- and M246-2-treated groups on Day 3 and Day 6 after fertilization. On Day 9, the relative abundance of Kosakonia in the GXGL-4A and M246-2 treatment groups was statistically significant (*p* < 0.01), but there was no significant difference between the GXGL-4A and M107 treatment groups.

The abundance of Kosakonia in each group was analyzed. The results indicated that the abundance of the genus Kosakonia varied at three sampling time points after the application of NFB cells. On Day 3, the abundance of Kosakonia in the M107 treatment group was always higher than that in the GXGL-4A treatment group, and the relative abundance of the M246-2 treatment group was the lowest. The abundance ratio of the GXGL-4A treatment group was 2.359%, while that of the M107 treatment group was 8.123% (Figure S2A). Before Day 6, the abundance of Kosakonia in the M107 treatment group was significantly higher than that in the 4A and M246-2 groups (*p* < 0.05) (Figure S2B). On Day 9, the abundance of Kosakonia in the M246-2 group was significantly lower than that in the GXGL-4A treatment group (*p* < 0.05), and in the GXGL-4A treatment group, the abundance of Kosakonia increased first and then decreased (Figure S2C).

The application of NFB not only affected the content of nitrogen and enzyme activity in the cucumber rhizosphere soil, but also significantly reshaped the microbial community composition and diversity. Through environmental factor correlation analysis at the genus level, it was found that NFB strains affected the microbial communities by altering the soil total nitrogen (TN) content. Among the top fifty dominant genera in terms of abundance, only nine genera, such as Hephaestia, Hyphomicrobium and Steridobacterium, showed no correlation with the tested environmental factors. The total nitrogen (TN), soil dehydrogenase (S-DHA) and soil ammonium nitrogen (NH_4_^+^-N) were the three key environmental factors, which showed significant correlations with the abundance of the most dominant genera in the rhizosphere soil. Secondly, the nitrate nitrogen (NO_3_^−^-N) and nitrite nitrogen (NO_2_^−^-N) contents were most negatively correlated with the abundance of the dominant genera. The S-POD and S-CL activities had a negligible effect on the abundance of the soil’s dominant microbial flora (Figure S3).

The results of a two-way correlation network analysis indicated that at the genus level, there was a networked interaction between the environmental factors evaluated and the abundance of bacterial species in the cucumber rhizosphere soil. Alterations in the soil nitrogen content and dehydrogenase activity would reshape the composition and abundance of the soil microbiota (Figure S4).

## 4. Discussion

### 4.1. Contrast to Prior Research

A siderophore-deficient mutant M246-2 was obtained from the Tn5 mutant library of *K*. *radicincitans* GXGL-4A using a CAS assay method in this study, and except for this, the WT GXGL-4A and high-siderophore-yielding mutant M107 showed strong CAS reactivity, indicative of siderophore production. Siderophores produced by these NFB strains are essential for iron acquisition and consequently modulate the relative growth rates of bacterial cells. In general, the siderophore-deficient mutant M246-2 grew the slowest in media supplied with limited iron (0–4 µmol/L) compared to other strains. Biofertilizers have been widely used in agricultural production because they can effectively promote plant growth. The reasonable addition of plant growth-promoting rhizobacteria (PGPR) can also regulate soil nutrients and prevent plant diseases by hindering the proliferation of pathogenic bacteria [25]. It has been confirmed that biological fertilizer can affect the rhizosphere microbial community and plant growth, but the effect of NFB as biofertilizer on the bacterial community of cucumber rhizosphere soil is unclear [26]. Currently, the development of high-throughput sequencing technology and bioinformatics technology makes it possible to study the bacterial community structure quickly and easily in different habitats. 16S rRNA gene sequencing is a common tool in microbiome investigation, and it is still being improved to increase its sensitivity and applicability in environmental bacterial population diversity analysis, which is driving the rapid progress of modern agricultural research [27].

A previous study reported that *Bacillus subtilis* and *Bacillus licheniformis* treatments increased the *α*-diversity of the maize rhizosphere soil bacterial community, and different fertilization treatments affected the abundance and diversity of the soil bacterial community [28]. Our study results were consistent with this report. The application of NFB strains significantly modified the bacterial community in the rhizosphere soil of cucumber. There was a significant difference in the *α*-diversity between the GXGL-4A and M107 treatment groups (*p* < 0.05), indicating that the synthesis capability for siderophores of the NFB GXGL-4A could affect the reshaping of the bacterial community in cucumber rhizosphere soils through a short-term fertilization. However, there was no significant difference in the soil bacterial community between the GXGL-4A and M246-2 treatment groups. Fertilization with the mutant M107 significantly reduced the abundance and diversity of bacterial communities in the rhizosphere soil compared to the other groups.

There was a higher abundance of the genera Arthrobacter, Bacillus and Kosakonia in the rhizosphere soil after the applications of GXGL-4A, M107 and M246-2 bacterial cells. Many bacterial strains in the genus Arthrobacter can degrade nitroglycerin, benzene derivatives, polycyclic aromatic compounds, halogenated alcohols, halogenated hydrocarbons, N-heterocyclic compounds, pesticides and herbicides [29,30,31,32,33,34]. Thus, NFB fertilization theoretically could promote plant growth by way of the degradation of toxic compounds in the soil.

A variety of strains in the genus of Kosakonia can promote plant growth by producing auxin and siderophores and increasing the phosphorus content [35]. In our study, the relative abundance of Kosakonia varied in different groups. At the same sampling time point, the relative abundance of Kosakonia in the M07 group was significantly higher than that in the GXGL-4A-treated group, and similarly, the abundance of Kosakonia in the GXGL-4A treatment group was significantly higher than that in the M246-2 fertilization group (*p* < 0.05). Therefore, we speculated that the activity of siderophore production is closely related to the survival status of a certain bacterium in the soil.

The soil enzymatic activity reflects the strength of the soil substance transformation (e.g., to C, N and P) and is suitable as an indicator parameter for measuring the effectiveness of fertilization [36]. The S-CL and S-DHA enzymatic activities can indicate the soil fertility well. In this work, the enzymatic activities of S-CL and S-DHA in cucumber rhizosphere soil were measured at three sampling time points (i.e., Day 3, Day 6 and Day 9 after NFB fertilization). Soil cellulase (S-CL) is an important enzyme in the carbon cycle of soil [37,38]. In general, the S-CL enzyme activity of the three NFB treatment groups showed a trend of first increasing and then decreasing. On Day 6 after NFB treatment, the S-CL enzyme activity was the highest, and the three S-CL enzyme activities of the NFB treatment groups were significantly higher than that of the CK group (*p* > 0.05). These results indicated that after the application of NFB, the S-CL enzyme activity in the rhizosphere soil was greatly increased, which accelerated the decomposition of cellulose in the soil to provide a sufficient carbon source for the absorption and utilization of cucumber seedlings, which was conducive to promoting their vegetative growth. The S-DHA activity has been used as a sensitive indicator to monitor respiratory and biochemical processes in soil microbial communities [39]. In the present study, the enzymatic activities in the GXGL-4A and M107 treatment groups increased sharply on Day 3 and Day 9, and the tested soil enzymatic activities were significantly higher than those of the other two groups, suggesting that the soil respiration strength was significantly enhanced and the nitrogen cycling in the soil was improved. After the treatment with the bacterial cells of siderophore-producing mutants, the tested soil enzyme activities significantly varied, which affected the nitrogen cycle of the cucumber rhizosphere soil. 

The soil inorganic nitrogen content has a direct impact on the crop growth, yield and quality, so it is regarded as an evaluation index for nutrient management in the field [40]. In this study, the three NFB biofertilizers could significantly increase the contents of the TN, NH_4_^+^-N and NO_2_^−^-N in cucumber rhizosphere soils. We speculate that the NFB strain GXGL-4A may also stimulate cucumber roots to secrete specific substances to recruit beneficial bacterial populations, thereby promoting cucumber growth. As for how the inoculation of GXGL-4A changes the composition and structure of cucumber root exudates, it will be one of the future research topics. In addition, a growing body of research indicates that the microbial composition is largely determined by environmental factors [41,42]. The differences in the siderophore-producing abilities of the NFB mutants affected the iron absorption capabilities of the bacteria themselves and the rhizosphere soil microbiota, modulated the utilization efficiency of soil microorganisms for nitrogen and iron nutrients, and thus reshaped the rhizosphere soil bacterial communities.

### 4.2. Implications of Research Results

The promoting effect of associative NFB strains on plant growth has been widely confirmed by the scientific community. In addition to the effectiveness of biological nitrogen fixation (BNF) in enhancing soil fertility, NFB cells competitively extract iron from the soil by secreting siderophores to inhibit soil-borne phytopathogens and promote plant growth, which is also an important component of their growth-promoting mechanism. The application of the high-siderophore-yielding mutant M107 showed a better growth promoting effect on cucumber plants. In an A15 nitrogen-free medium supplemented with different concentrations of iron nutrition, M107 grew faster, indicating that it could better obtain ferric nutrition from the external environment. The ability to produce siderophores is also closely related to the colonization ability of the bacterium in the soil environment. The abundance of Kosakonia species was highest in the soil of the M107 treatment group, suggesting that the mutant had a stronger survival ability compared to the WT strain GXGL-4A and the siderophore synthesis-deficient mutant M246-2.

In current agricultural production, the yield improvement effect of NFB agents applied in the field is often unstable. This is not only related to environmental factors such as ultraviolet radiation, high temperatures and drought stress, but is also closely attributed to the deterioration of the soil microecology and the complex interactions between NFB, plants and soil microbial communities. Therefore, it is important to investigate the effects of NFB fertilization on the rhizosphere microbiota of plants, which is also one of the research hotspots in the field of microbial ecology. Our research results indicate that the application of a high-siderophore-yielding NFB agent can significantly promote the growth of cucumbers and improve the soil microecological environment by significantly increasing the activities of the soil enzymes S-POD and S-DHA and altering the soil nitrogen levels.

## 5. Conclusions

The application of NFB strains with different siderophore-producing capabilities (i.e., GXGL-4A, M107 and M246-2) can significantly promote plant growth and reshape the rhizosphere soil microecology of cucumber. The stronger the ability of NFB to produce siderophores, the greater the impact on the microecology, including the enzymatic activities of S-POD and S-DHA, the soil NH_4_^+^-N and TN contents and the bacterial community composition and abundance. The highest abundance of Kosakonia bacteria in the rhizosphere soils treated with the mutant M107 with an enhanced ability to produce siderophores was detected in this study, suggesting that this mutant has the strongest survival ability in soils compared to GXGL-4A and M246-2. The inoculation of cucumber plants with the NFB strain GXGL-4A and its mutants could significantly affect the abundance of the dominant phyla, such as Actinobacteriota, Bacteroidota and Patescibacteria, as well as the abundance of the dominant genera, such as Devonia, Streptomyces and Arthrobacter. Overall, the changes in the soil nitrogen contents indicate the diversity and abundance of the rhizosphere microbiota well with the application of an NFB biofertilizer, while the activity of most soil enzymes (including S-CL and S-POD) is not suitable as an indicator for the profiling of the rhizosphere bacterial community. The ability of NFB to synthesize siderophores determines their adaptability to the soil environment and the strength of their regulatory effect on the rhizosphere soil microecology. Further research may focus on the regulation mechanism of siderophore biosynthesis in the NFB strain GXGL-4A and the response of the plant root transcriptome to NFB treatment, in order to further explore the interaction between NFB and plants.

## Figures and Tables

**Table 1:** The abundance and diversity indices of the bacterial community in the NFB treatment cucumber rhizosphere soils. The soil samples of the CK group sampled on Day 3, Day 6, and Day 9 after sterile water treatment are represented by CK-T1, CK-T2 and CK-T3, respectively. Soil samples sampled on Day 3, Day 6 and Day 9 after GXGL-4A treatment are recorded as 4A-T1, 4A-T2 and 4A-T3, respectively. Soil samples sampled on Day 3, Day 6 and Day 9 after M107 treatment are shown as M107-T1, M107-T2 and M107-T3, respectively. Soil samples sampled on Day 3, Day 6 and Day 9 after GXGL-4A treatment are recorded as 4A-T1, 4A-T2 and 4A-T3, respectively. Sobs, Chao 1, Shannon and Ace indices based on the OTU level are shown. Lowercase letters indicate that there was a significant difference in the *α*-diversity index between two groups according to a *t*-test (*p* < 0.05).

	Treatments	Ace Index	Chao 1 Index	Shannon Index	Sobs
Day 3					
	CK-T1	3607.64 ± 524.56 b	3492.49 ± 439.99 b	6.07 ± 0.32 b	2680.67 ± 366.85 b
	4A-T1	4844.78 ± 307.74 a	4616.06 ± 227.80 a	6.56 ± 0.08 a	3522.67 ± 169.09 a
	M107-T1	5204.10 ± 199.79 a	4967.41 ± 178.73 a	6.50 ± 0.20 a	3710.67 ± 164.81 a
	M246-2-T1	4824.56 ± 144.89 a	4524.58 ± 158.61 a	6.56 ± 0.16 a	3507.33 ± 131.96 a
Day 6					
	CK-T2	4167.03 ± 345.49 ab	3976.52 ± 310.54 ab	6.03 ± 0.31 a	2913.67 ± 277.03 ab
	4A-T2	4007.96 ± 338.46 b	3862.98 ± 305.97 b	6.05 ± 0.09 a	2807.00 ± 189.61 b
	M107-T2	4760.31 ± 6.17 a	4514.90 ± 53.70 a	6.35 ± 0.03 a	3337.33 ± 55.79 a
	M246-2-T2	4340.41 ± 450.89 ab	4067.11 ± 357.73 ab	6.24 ± 0.11 a	3081.33 ± 276.96 ab
Day 9					
	CK-T3	5197.03 ± 181.39 a	4910.15 ± 194.28 a	6.63 ± 0.03 a	3703.67 ± 80.75 a
	4A-T3	4887.19 ± 249.69 ab	4595.86 ± 210.80 ab	6.45 ± 0.23 ab	3444.00 ± 203.74 ab
	M107-T3	4531.32 ± 292.67 b	4302.62 ± 257.56 b	6.28 ± 0.19 b	3193.00 ± 229.69 b
	M246-2-T3	5179.48 ± 125.12 a	4884.79 ± 45.97 a	6.74 ± 0.05 a	3751.67 ± 67.72 a

## Data Availability

The original contributions presented in this study are included in the article/Supplementary Materials; further inquiries can be directed to the corresponding author. The 16S rRNA gene amplicon sequencing data of the cucumber rhizospheres treated, respectively, with the wild-type strain GXGL-4A and the mutants M246-2 and M107 have been deposited in the Sequence Read Archive (SRA) database with the BioProject number PRJNA1072636.

## Supplementary Materials

The following supporting information can be downloaded at: https://www.mdpi.com/article/10.3390/microorganisms13020346/s1. Figure S1: Significant differences were analyzed at the phylum (A) and genus (B) levels between treatment groups. One asterisk (*) indicates a significant difference in the relative abundance of microbial species (*p* < 0.05). The multiple testing correction method used for *p* values was the false discovery rate (FDR). Figure S2: Difference test analysis of samples at Kosakonia genus level. Kruskal–Wallis H test was used for significance testing among soil sample groups. Figure S3: Spearman correlation heatmap at the genus level. An asterisk indicates a significant correlation between an environmental factor and the abundance of a species in the genus (*p* < 0.05). Similarly, two or three asterisks indicate a highly significant correlation between an environmental factor and the abundance of a species in that genus (*p* < 0.01 and *p* < 0.001, respectively). The top 50 genera in terms of abundance are displayed in descending order in the figure. Figure S4: Network diagram of the correlation between bacterial species and environmental factors at the genus level. The red and green lines, respectively, represent the positive and negative correlations between environmental factors and the abundance of a certain genus species. The thicker the line, the stronger the correlation.
