# Supplementary figures and images for: Siderophore Production Capability of Nitrogen-Fixing Bacterium (NFB) GXGL-4A Regulates Cucumber Rhizosphere Soil Microecology
Source: Microorganisms. 2025 Feb 5;13(2):346. doi: 10.3390/microorganisms13020346 (PMC11858475; doi:10.3390/microorganisms13020346)

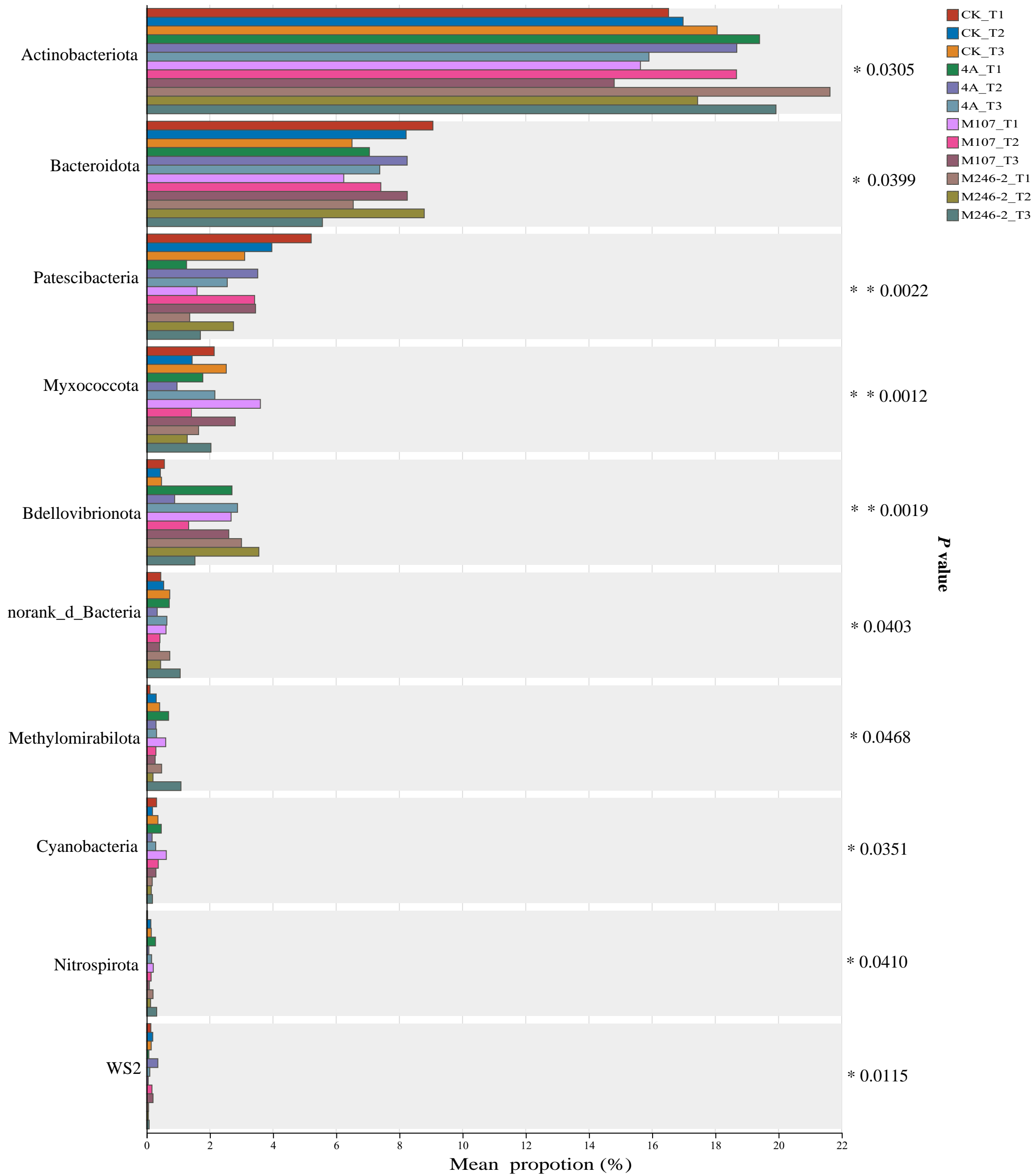

Supplement: Supplementary file 1 [file microorganisms-13-00346-s001.zip › Supplementary files/Fig. S1A (at phylum level).pdf]

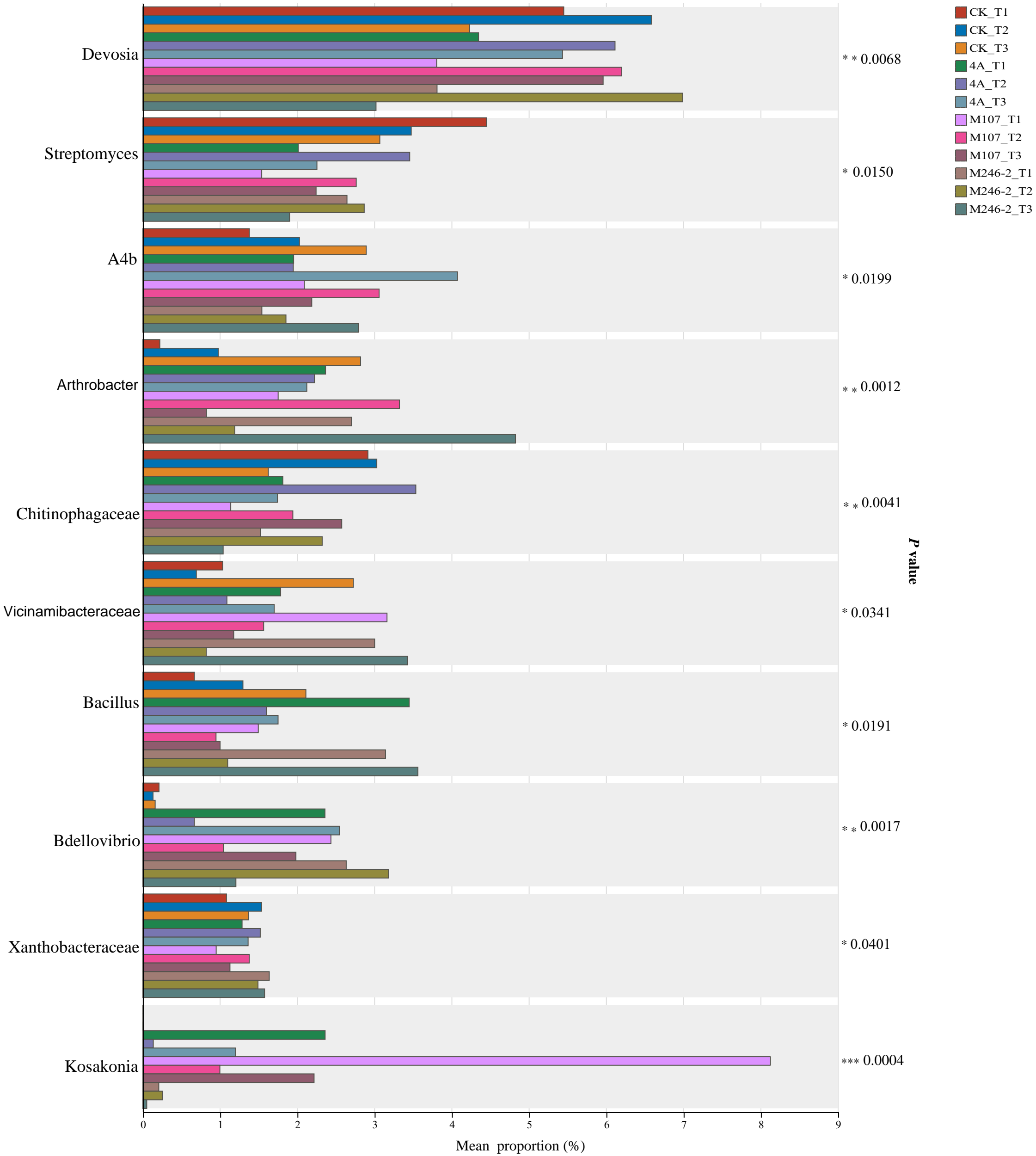

Supplement: Supplementary file 1 [file microorganisms-13-00346-s001.zip › Supplementary files/Fig. S1B (at the genus level).pdf]

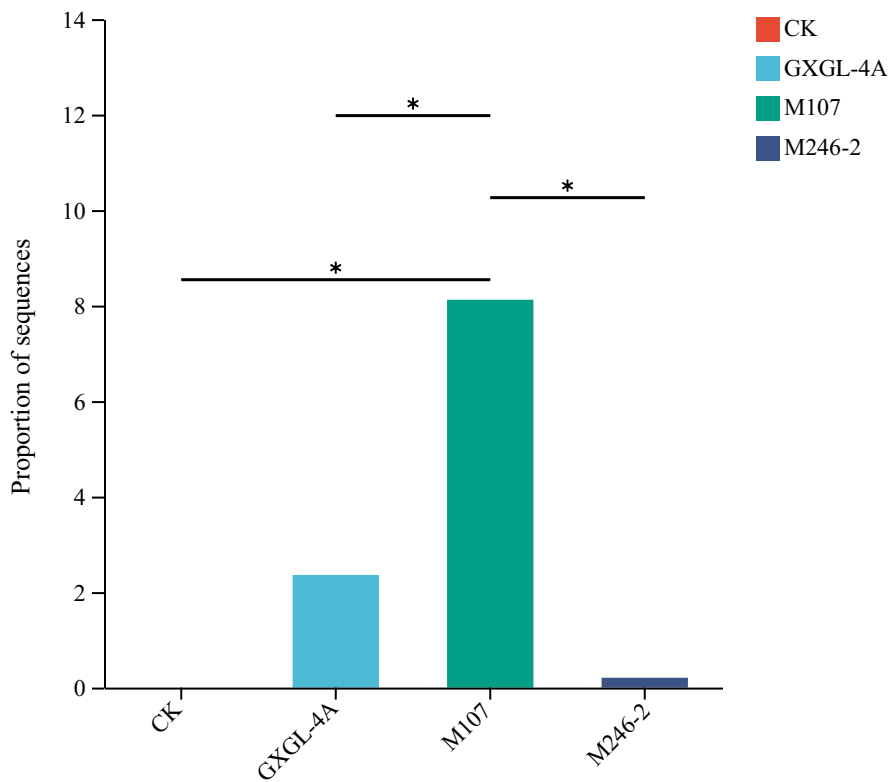

Supplement: Supplementary file 1 [file microorganisms-13-00346-s001.zip › Supplementary files/Fig. S2A.pdf]

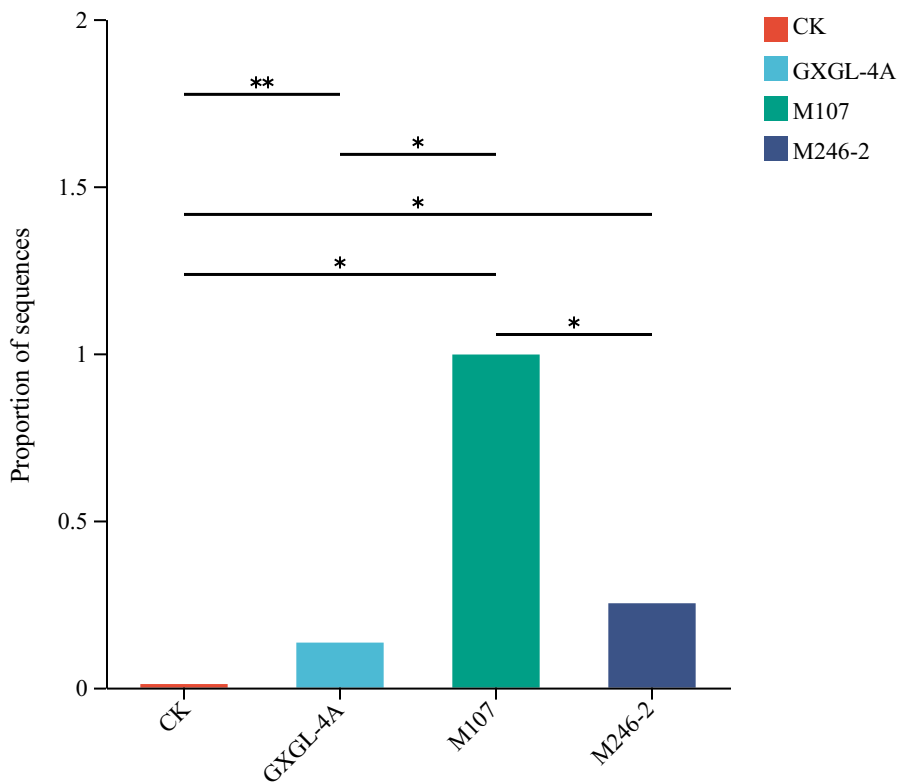

Supplement: Supplementary file 1 [file microorganisms-13-00346-s001.zip › Supplementary files/Fig. S2B.pdf]

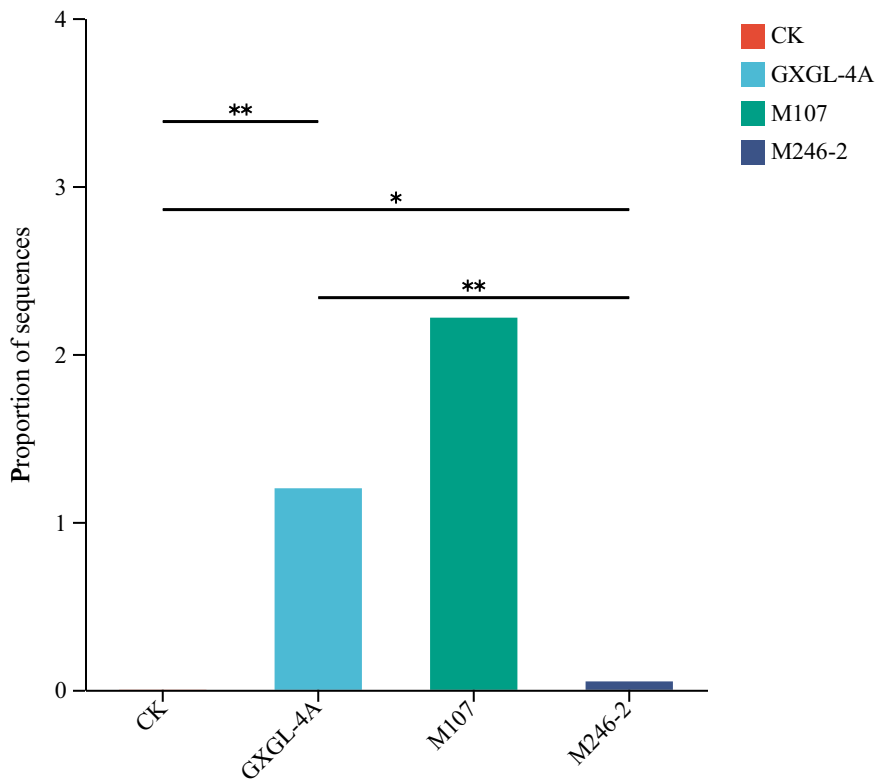

Supplement: Supplementary file 1 [file microorganisms-13-00346-s001.zip › Supplementary files/Fig. S2C.pdf]

Spearman Correlation Heatmap

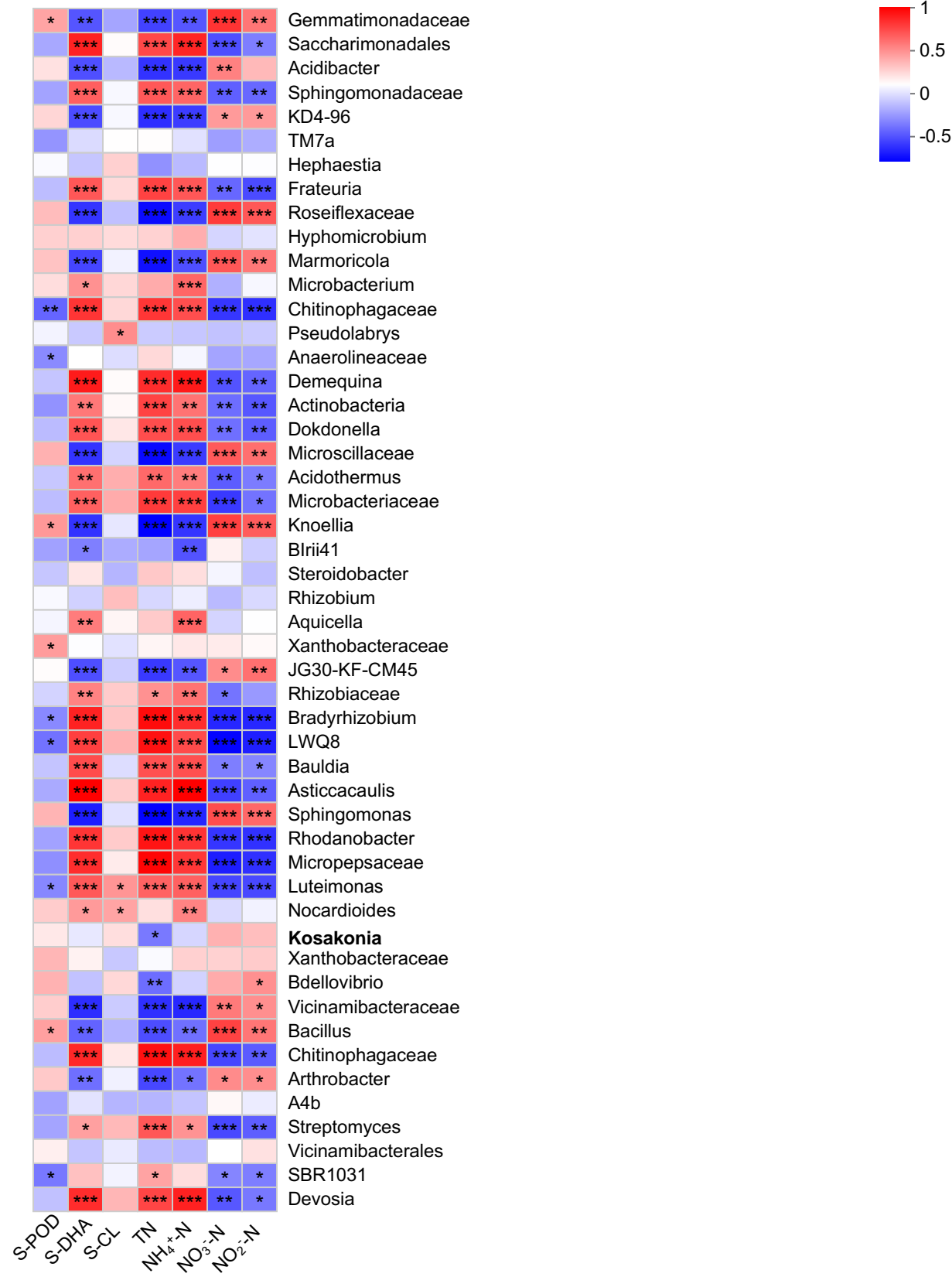

Supplement: Supplementary file 1 [file microorganisms-13-00346-s001.zip › Supplementary files/Fig. S3 correlation heatmap (at genus level).pdf]

# Network analysis

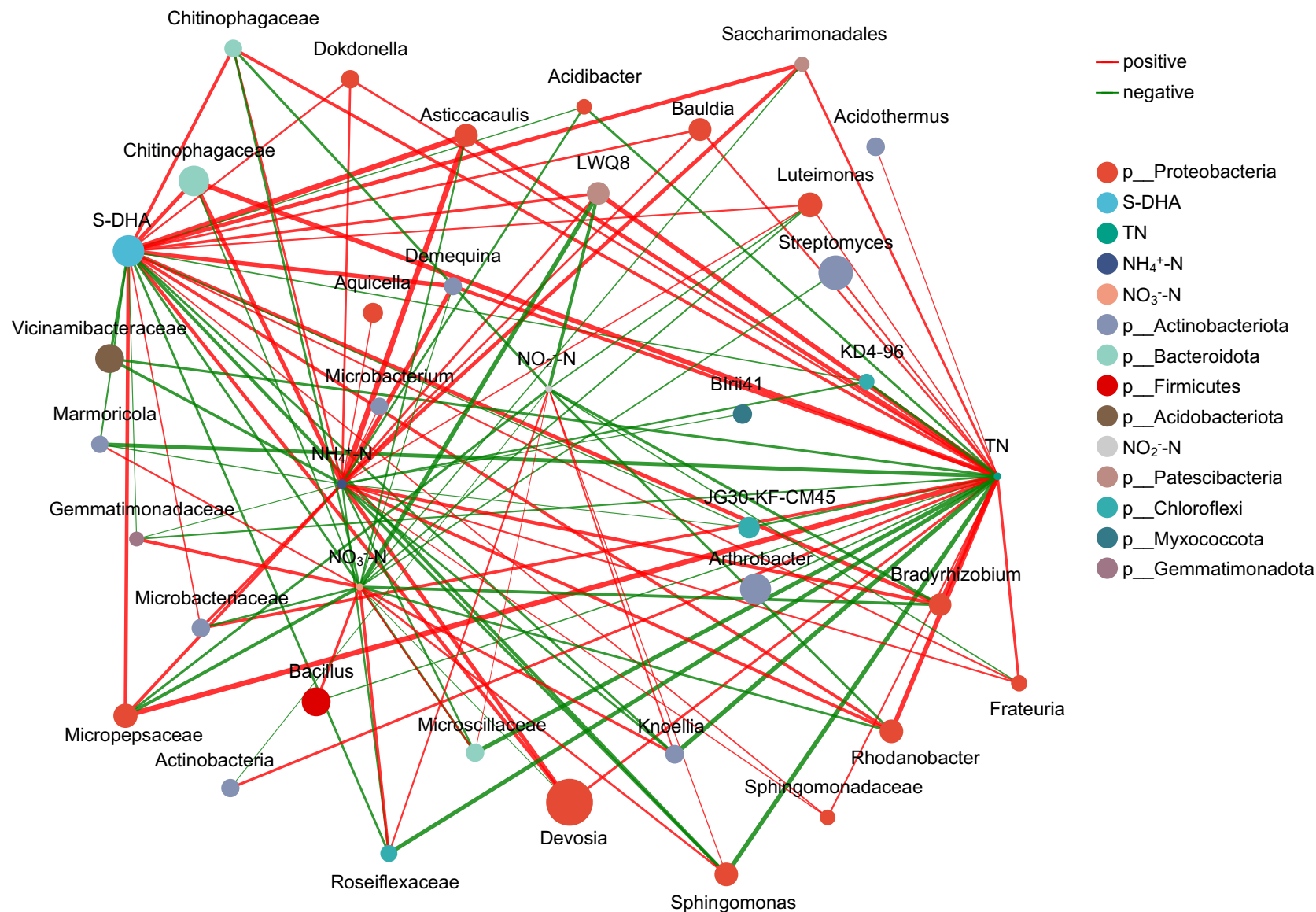

Supplement: Supplementary file 1 [file microorganisms-13-00346-s001.zip › Supplementary files/Fig. S4 correlation network(at genus level).pdf]
